# Experimental Muscle Pain Impairs the Synergistic Modular Control of Neck Muscles

**DOI:** 10.1371/journal.pone.0137844

**Published:** 2015-09-18

**Authors:** Leonardo Gizzi, Silvia Muceli, Frank Petzke, Deborah Falla

**Affiliations:** 1 Pain Clinic, Center for Anesthesiology, Emergency and Intensive Care Medicine, University Hospital Göttingen, Göttingen, Germany; 2 Department of Neurorehabilitation Engineering, Bernstein Center for Computational Neuroscience, University Medical Center Göttingen, Georg-August University, Göttingen, Germany; Universite de Nantes, FRANCE

## Abstract

A motor task can be performed via different patterns of muscle activation that show regularities that can be factorized in combinations of a reduced number of muscle groupings (also referred to as motor modules, or muscle synergies). In this study we evaluate whether an acute noxious stimulus induces a change in the way motor modules are combined to generate movement by neck muscles. The neck region was selected as it is a region with potentially high muscular redundancy. We used the motor modules framework to assess the redistribution of muscular activity of 12 muscles (6 per side) in the neck region of 8 healthy individuals engaged in a head and neck aiming task, in non-painful conditions (baseline, isotonic saline injection, post pain) and after the injection of hypertonic saline into the right splenius capitis muscle. The kinematics of the task was similar in the painful and control conditions. A general decrease of activity was noted for the injected muscle during the painful condition together with an increase or decrease of the activity of the other muscles. Subjects did not adopt shared control strategies (motor modules inter subject similarity at baseline 0.73±0.14); the motor modules recorded during the painful condition could not be used to reconstruct the activation patterns of the control conditions, and the painful stimulus triggered a subject-specific redistribution of muscular activation (i.e., in some subjects the activity of a given muscle increased, whereas in other subjects it decreased with pain). Alterations of afferent input (i.e., painful stimulus) influenced motor control at a multi muscular level, but not kinematic output. These findings provide new insights into the motor adaptation to pain.

## Introduction

Neck pain is associated with changes in cervical sensorimotor control. For instance, individuals with neck pain may display a loss of neck muscle directional specificity [1,2] and increased co-activation of neck flexor and extensor muscles during functional tasks [3,4]. In addition, acute pain induces an immediate change in muscular motor control [5–9]. Although these studies confirm that pain is associated with altered muscle activation, the changes observed are highly variable, can be contradicting and rarely fit with the explanation offered by simple theories of motor adaptation to pain [10,11]. However, earlier studies have classically evaluated the effect of neck pain on a small number of synergistic and antagonistic muscles which limits the interpretation of the effect of neck pain on the neural control of neck movement [12]. A more comprehensive analysis of the redistribution of muscle activity with pain is warranted.

Pain-induced changes in muscle activation are often accompanied by changes in motor output, such as decreased maximum force [13], decreased force steadiness [14], decreased range of motion [15] and decreased movement speed [16]. On the other hand, for conditions with relatively low mechanical demand, the kinematic output may not change and the task is executed in the same manner in the presence of pain [9,17,18]. This is likely due to redistribution of activity among synergist and antagonist muscles in regions with high redundancy. The neck is a suitable example of such a region, since several neck muscles have similar lines of action [19] and therefore different muscles can conceivably be recruited to perform the same task.

Although the redundancy of motor actuators in the body (from individual motor units to joints) potentially allows the central nervous system (CNS) to accomplish goal-directed tasks with different combinations of muscle recruitment, in normal conditions the CNS chooses solutions that involve combinations of a small number of motor modules, also referred to as muscle synergies [20].The presence of regularities in motor control is universally acknowledged, although whether those structures reflect neural encoding or emerge from biomechanical constrains is still debated (for a comprehensive review see [21]). Several studies support the notion that the CNS can achieve the control of the numerous degrees of freedom of the body by means of a reduced set of activation signals and motor modules [22–25]. Motor modules have been hypothesized to decrease the computational burden of movement, distribute the management of the actuators activity to different neural levels or represent a preferred channel from which motor commands can be specified when a task that could be performed following different trajectories, is executed [26]. In a wider perspective they can be seen as the function translating the CNS commands from the “task” level (e.g., moving from point A to point B, reaching an object, or maintain balance) to the required muscular excitation pattern [27]. The hierarchical organization of motor modules has also been associated with those parts of the CNS that are phylogenetically more primitive [28–30]. It is likely that motor modules incorporate an internal model of the musculoskeletal system, which dynamically improves during the development of the individual, refining the control of movement and adjusting to changes of the biomechanical properties of the body [31–33]. It has been shown that the motor modules are recruited in a flexible task and constraint-dependent manner in animals [22] and humans during reaching and locomotion [23,34–37]. Furthermore, recent evidence suggests that altered afferent feedback may modify existing motor modules or lead to the recruitment of new ones [17,38–40].

On the other hand through cadaveric experiments and computational models, it has been shown that task constrains and/or the biomechanics of joints itself may result in the appearance of a modularity of control even if the muscles are governed individually [41]. In other words the more biomechanical constrains exist to a task, the smaller is the space of solutions the CNS can retrieve the actual motor pattern from.

In this study, we evaluate 1. whether the control of neck muscle activity during multi-directional, multi-planar aiming movements of the head, can be characterized by motor modules and 2. the effect of experimentally induced neck muscle pain on the activation of multiple neck muscles during these movements. To characterize motor control at a multi-muscular level, the activation patterns of the investigated muscles were reconstructed by means of a model based on time-invariant motor modules and activation signals [22]. It was hypothesized that experimental neck muscle pain would alter the activation of the muscle at the site of noxious stimulation [17,42] and, as a consequence, induce a reorganization of the modular control of neck movement.

## Materials and Methods

### Subjects

Eight young, healthy individuals (age: 24.1±1.9 years, stature: 171.6±14.7 cm, weight: 65.6±16.0 kg) volunteered for this study. Participants were excluded if they had a history of neck or back pain which required treatment from a health professional, any major circulatory, neurological, respiratory disorders, recent/current pregnancy, or previous spinal surgery. Ethical approval for the study was granted by the Ethics Committee of Universitätsmedizin Göttingen, Germany and the procedures were conducted according to the Declaration of Helsinki. The participants provided written informed consent and the Ethics Committee of Universitätsmedizin Göttingen, Germany approved the informed consent procedure.

### Experimental procedure

**Fig 1:**
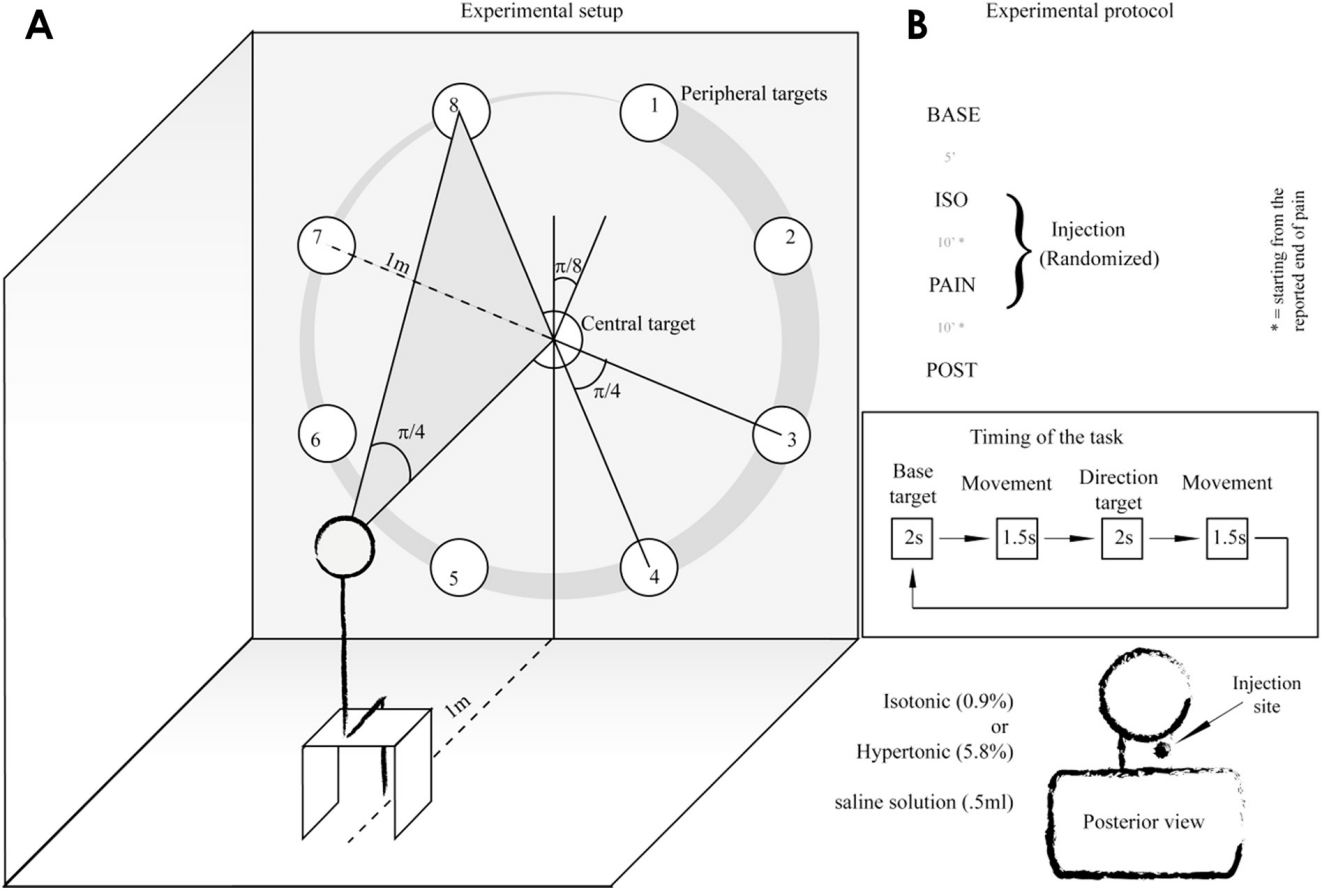
Experimental setup and protocol. A. schematic representation of the target distribution: targets were aimed either in a clockwise or counterclockwise order, the numbers are indicative of the clockwise case. B: conditions sequence (top), aiming timing within the same condition (center) and injection specifications (bottom).

Participants performed multi-directional, multi-planar aiming movements of the head (Fig 1). Nine circular targets (20 cm diameter; one “central target” plus 8 “peripheral targets”) were placed on a whitewall following a circular trajectory. The central target was placed in the center of an ideal circle of 1m radius and the peripheral targets were equally distributed (π/4 apart) with an offset of π/8 from the vertical line.

Subjects were seated on a backless chair. They wore a custom-made helmet equipped with a symmetrically distributed weight (600g) and two laser pointers (<1mW) mounted parallel to the vision line. The seat height was adjusted so that the laser pointer line matched the central target.

The task consisted of moving the head and neck to aim the laser pointers from the central target to each peripheral target (in a clockwise or a counterclockwise sequence; randomized) following the tempo provided by a metronome. Subjects were asked to perform the movement (center to periphery) in 1.5 s, maintain the laser beams on the peripheral target for 2 s, return to the central target over 1.5 s and then rest at the central target for 2 s before moving to the next peripheral target. Thus, the total duration was approximately 56 s per turn (the entire sequence was repeated twice without rest). Subjects were instructed to perform the movement as precisely as possible and avoid corrective actions once the target was reached.

In an attempt to prevent effects due to learning, the subjects practiced the task until they were accustomed to it prior to the recordings (~5 minutes). The task was performed in four conditions: at baseline (BASE condition), immediately after the injection of isotonic (ISO condition) and hypertonic saline (PAIN condition), and 10 min after the painful sensation induced by the saline injection disappeared (POST condition). The POST condition served to exclude adaptation or learning. A rest period of ~5 min was given following the BASE condition and 10 min was given between injections. The injection sequence was randomized. Participants were blinded to each injection and were told that one or both might be painful.

### Experimental muscle pain

Experimental muscle pain was induced by injection (27-gauge cannula) of 0.5 ml of sterile hypertonic saline (5.8%) into the right *Splenius Capitis* muscle as previously described (9). Isotonic saline (0.5 ml, 0.9%) was used as a control injection in the same muscle. The experimental task started immediately after the injection in both ISO and PAIN conditions. Subjects were asked to verbally report their level of pain intensity on a eleven point numerical rating scale (NRS) ranging from 0 (no pain perceived) to 10 (worst pain imaginable) every 30 s starting from the extraction of the needle until pain vanished. After the subjects reported 0 value, an additional pain report was asked after 30 s.

### Motion detection

Tridimensional tracking of head and shoulder movement was achieved by means of an eight camera stereo-photogrammetry system (Oqus 300+, Qualisys Gothenburg, Sweden) at 64 frames per second. Three markers were placed on the helmet (front, right side, back), and two markers over the subject’s acromia (RSHO and LSHO). One of the channels from the electromyography (EMG) amplifier (see below) was mirrored on the analog board of the motion capture system to allow synchronization. Analog data was sampled at 2048 samples per second (12 bit resolution) and stored together with the three dimensional data from the markers. The time delay between the motion capture and EMG systems was estimated by means of the global maximum of the cross correlation function between the original and the mirrored EMG [37].

Kinematic data from the helmet was used to detect movement of the subject’s head from the center to peripheral targets and vice versa. The module of velocity was calculated for each of the three markers and then averaged. A threshold of 5% of the maximal speed for each direction was used for segmentation. Movement duration, maximal speed in each segment, time to peak (i.e. the time to reach the maximal speed) and distance traveled were computed for each direction in each condition for all subjects.

Subjects were asked to perform the task with minimal movement of the trunk; to verify this, the average distance traveled was also computed for the shoulder markers. For all subjects the distance traveled by the shoulders never exceed 10% of the distance traveled by the helmet markers.

### Electromyography

Surface EMG signals from bipolar derivation (Ambu Neuroline 720 01-K/12; Ambu, Ballerup, Denmark) were recorded from 12 muscles (six per body side) in the neck region (*Sterno Hyoideus*–HYO, *Sternocleidomastoideus*-STER, *Anterior Scalenus-*SCA, *Splenius Capitis*–SPL, *Upper trapezius*-UTR, *Lower Trapezius-*LTR). The electrodes were positioned according to guidelines for electrode placement [43,44]. Data was digitally converted and sampled at 2048 samples per second (12 bits per sample, 8^th^-order Bessel filter, bandwidth 10–450 Hz, EMG-USB2, OT Bioelettronica, Torino, Italy) and stored on a computer’s hard disk drive for further analyses.

After synchronization with motion capture, surface EMG data was segmented (based on the helmet velocity) in order to obtain muscular activation during the transition from center to periphery and vice versa. The EMG segment for each movement started 100 ms before the velocity reached a threshold of 5% of the maximal speed of the movement and ended 100 ms after the velocity signal crossed the threshold again. A non-negative matrix factorization algorithm (NMF) [45] was used to extract motor modules from the EMG signals. Data from one turn (i.e. 8 targets) in one condition for one subject was concatenated prior to filtering (with the aim of attenuating the cutting artifact [46]), rectified and low-pass filtered (zero lag Butterworth filter, 4^th^ order, with cut-off frequency of 1Hz [17,34]. Each movement was then resampled to 200 samples and the concatenation of the movements of one trial was used to perform motor module extraction [47]. Each condition was treated separately.

The signal used for the extraction of motor modules should carry enough information to capture the variability of the movement, but at the same time should be concise enough to represent a well-circumstantiated phase of the pain response. With this in mind, we verified that that one turn of movements provided enough data to obtain a reliable estimation of the motor modules: for the BASE condition we extracted the motor modules from the first and second turns and compared them in terms of similarity (see the Results section for details). Once it was assured that only one turn could be used, we focused on the pain profile evolution in time.

The pain perceived by the subjects following the injection of hypertonic saline rapidly increased and then decreased more slowly (see Results). The first turn for the painful condition was the one corresponding to the raising phase of pain, whereas the second one occurred when the pain was maximal. Therefore, only the second turn was considered for further analysis since the most dissimilar from the BASE condition. For consistency, only the second turn of the other conditions was also retained in the analysis

To test for the presence of a low dimensionality of motor control, muscle activation patterns were factorize to highlight structured content and to compare across different subjects and conditions. The extraction of motor modules was performed according to the following model:

$$X(k)={\left[x_{1}(k),x_{2}(k),\cdots ,x_{M}(k)\right]}^{T}$$ (1)

in which *X(k)* represents the activity of all muscles examined and *x*_*m*_ *(k)* represents the activity of the *m*th muscle at the time instant *k*.

*P(k)* are the activation signals, less than the number of muscles recorded (*N<M*) extracted via NMF:

$$P(k)={\left[p_{1}(k),p_{2}(k),\cdots ,p_{N}(k)\right]}^{T}$$ (2)

Muscle activity is obtained from the activation signals by linear transformation with weighting factors *s*_*mn*_. The matrix whose columns are the weights of each activation signal for each muscle is denoted as *S* and referred to as the matrix of motor modules [45]. The relation between *X(k)* and *P(k)* is described as follows:

$$X(k)\approx X_{r}(k)=S\cdot P(k)$$ (3)

where *X*_*r*_ *(k)* is the muscular activity reconstructed by means of motor modules and activation signals.

Modules were extracted according to the model in Eq (3). In the NMF algorithm the dimensionality of decomposition is not known *a priori*, therefore the extraction procedure was repeated for a number of modules ranging from 1 to 12, 12 being the number of muscles examined. In case of completely uncorrelated signals (e.g. white noise) the reconstruction quality (see below) would increase quasi-linearly whilst in case of a common underlying structure contaminated by uncorrelated signals (either noise or channel specific EMG) the reconstruction quality would increase non linearly and few factors (i.e. the motor modules) would be able to account for most of the information available.

The reconstruction quality was assessed by means of the Variance Accounted For (VAF) index defined as VAF = 1 –SSE/SST, where SSE (sum of squared errors) is the unexplained variance and SST (total sum of squares) is the total variance (of the data). A minimal VAF value of 95% was required for this study, while adding an additional synergy would not have increased the explained variance of more than 3% [36,48].

The NMF algorithm initiates from randomly generated non-negative matrices, thus the results may slightly vary at each run [45]. In order to minimize this possible bias, data was processed 10 times and the set of motor modules that showed the highest performance (i.e. highest reconstruction quality) was retained for further analyses [34].

The degree of similarity of motor modules across different conditions was assessed by means of the average normalized dot product (NDP) and by the cosine of principal angles (CPA) [24,49]. NDP measures the best-matching scalar product of two vectors of motor modules, divided by the product of the norms. This value ranges between 0 and 1 because the vectors are non-negative and equals 1 if they are matching perfectly, 0 if there is no correlation; NDP is a measure of the similarity of the structure of two modules. The CPA quantifies the degree of overlap between the subspaces that two sets of modules span and indicates if two sets of motor modules can generate the same spaces of muscular activation. A threshold of .8 was chosen to define two sets of muscle weightings “similar” for NDP and CPA(46). The similarity of activation signals for the same subject across conditions was calculated as the average of cross-correlation (zero lag) peak values of activation signal pairs associated with matched motor modules [46]. The clockwise sequence was arbitrarily selected as the default configuration, thus, the trials performed in counter-clockwise mode were rearranged before computing the cross correlation value.

Since no *a priori* hypotheses can be set on the level of stereotypy of motor response during aiming and its influence on modularity, in order to test the level of similarity of motor modules and activation signals across subjects, the NDP and cross correlation values were computed using the motor modules of a subject as a reference for a given condition; only the highest value was then retained.

In order to directly test if the muscle weightings extracted in one condition could describe the muscular pattern of another condition, the muscle activation pattern (EMG) of each subject in each condition was reconstructed by means of the matrix of motor modules extracted from another condition (cross reconstructions). For this analysis, a modified version of the NMF algorithm, referred in the following to as non-negative reconstruction [34] was used. The modules of one condition are retained and the activation signals are left free to vary while following the update rules of NMF.

The accuracy in reconstructing the muscular activation pattern with this procedure represents the maximal accuracy when using the matrix of motor modules from other conditions across all the possible choices of activation signals, with the only constraint of non-negativity. Similar VAF values would be expected in case of a common structure between the signal to reconstruct and the ones from which the original motor modules have been extracted.

EMG and kinematic data were processed with custom-made software written in Matlab code (Matlab 2010b, the Mathworks, Natick, Massachusetts).

### Statistical Analysis

Non-parametric statistics were applied since the data were not normally distributed. Friedman test for repeated measures was used to test the differences across conditions. When Friedman test showed significant differences, pair-wise comparisons were performed by means of Wilcoxon test for matched pairs. Statistical analysis was applied to kinematic (movement time, traveled distance, maximal speed, time to peak) and motor control variables (EMG amplitude, reconstruction quality, NDP and CPA with respect to baseline, cross correlation of the activation signals, cross-reconstruction).

The intensity and duration of pain in ISO and PAIN conditions were tested with Wilcoxon test for matched pairs. Statistical significance was set at P < 0.05.

## Results and Discussion

### Pain intensity

The pain caused by the two injections (isotonic and hypertonic saline) was different for intensity (P<0.001) and duration (P<0.001) (see Table 1). Subjects reported an average maximal value of 4.88 out of 10 following the injection of hypertonic saline.

**Table 1:** Mean and SD of pain intensity and pain duration reported following the injections. Pain intensity was computed as the average of all the pain intensity values during the performance of the experimental task.

	BASE	ISO	PAIN	POST
Pain intensity (NRS:0–10) ^	/	0.16(0.33)	3.59(0.74)	/
Pain duration (s) ^	/	4.25(9.74)	337.50(124.37)	/

^ For those subjects who reported pain only after the injection (but no pain after 30s), 1s was counted for average reasons. Zero seconds were considered for those subjects who reported no pain even immediately after the injection.

### Kinematics

**Fig 2:**
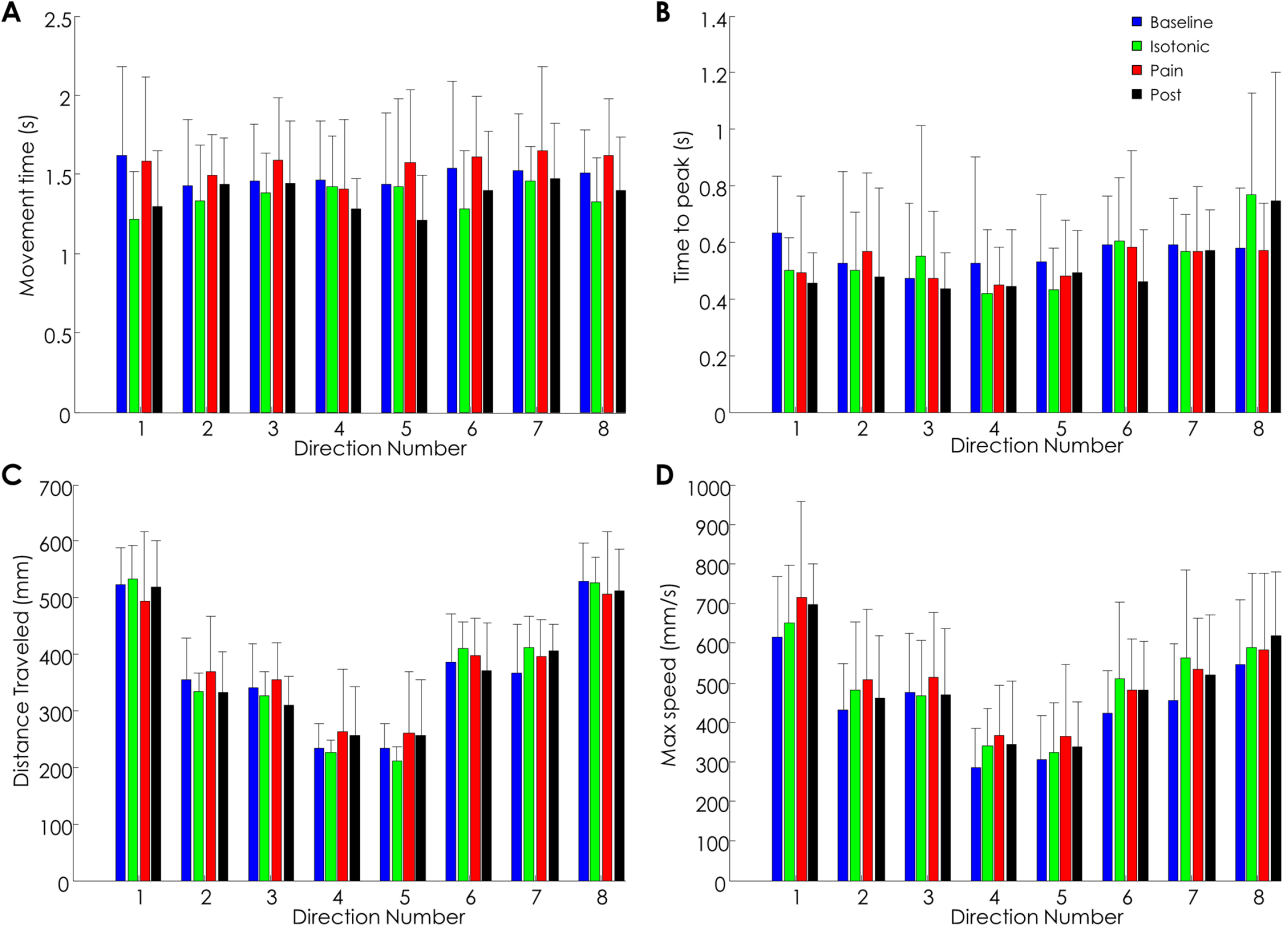
Kinematic data. Mean and standard deviation of the kinematic variables. A. movement duration; B. time to reach the peak speed; C. distance traveled; D. maximal speed. The distance traveled and maximal speed were modulated according to the biomechanical constraints of aiming in different directions; the movement duration and time to peak were invariant with respect to the combination of directions and conditions. Refer to Fig 1 for definition of the movement directions.

Despite the presence of pain, all subjects were able to accomplish the required task with no major precision or timing errors. The results of kinematic data analysis confirmed the general impression that the experimentally induced muscle pain did not affect the execution of the task (see Fig 2 and Table 2). In particular, the similarity in maximal speed and time to peak across the four conditions was likely due to the fact that movements were directed by auditory cues which helped the subjects to maintain the established pace, and throughout the entire experiment, the velocity profile was maintained bell-shaped for all the subjects.

**Table 2:** Results of the Friedman test (P values) for the comparison of kinematic variables across conditions: statistical analysis did not reveal any difference across conditions for all the considered kinematic variables in each movement direction (Dir). Refer to Fig 1 for definition of the movement directions.

Kinematic variable	Dir 1	Dir 2	Dir 3	Dir 4	Dir 5	Dir 6	Dir 7	Dir 8
Movement time (s)	0,05	0,14	0,25	0,58	0,43	0,22	0,43	0,62
Distance traveled (mm)	0,89	0,72	0,54	0,24	0,68	0,49	0,59	0,30
Time to peak (s)	0,41	0,25	0,89	0,86	0,80	0,62	0,54	0,99
Maximal speed (mm/s)	0,80	0,45	0,62	0,81	0,43	0,78	0,26	0,25

### EMG amplitude

**Fig 3:**
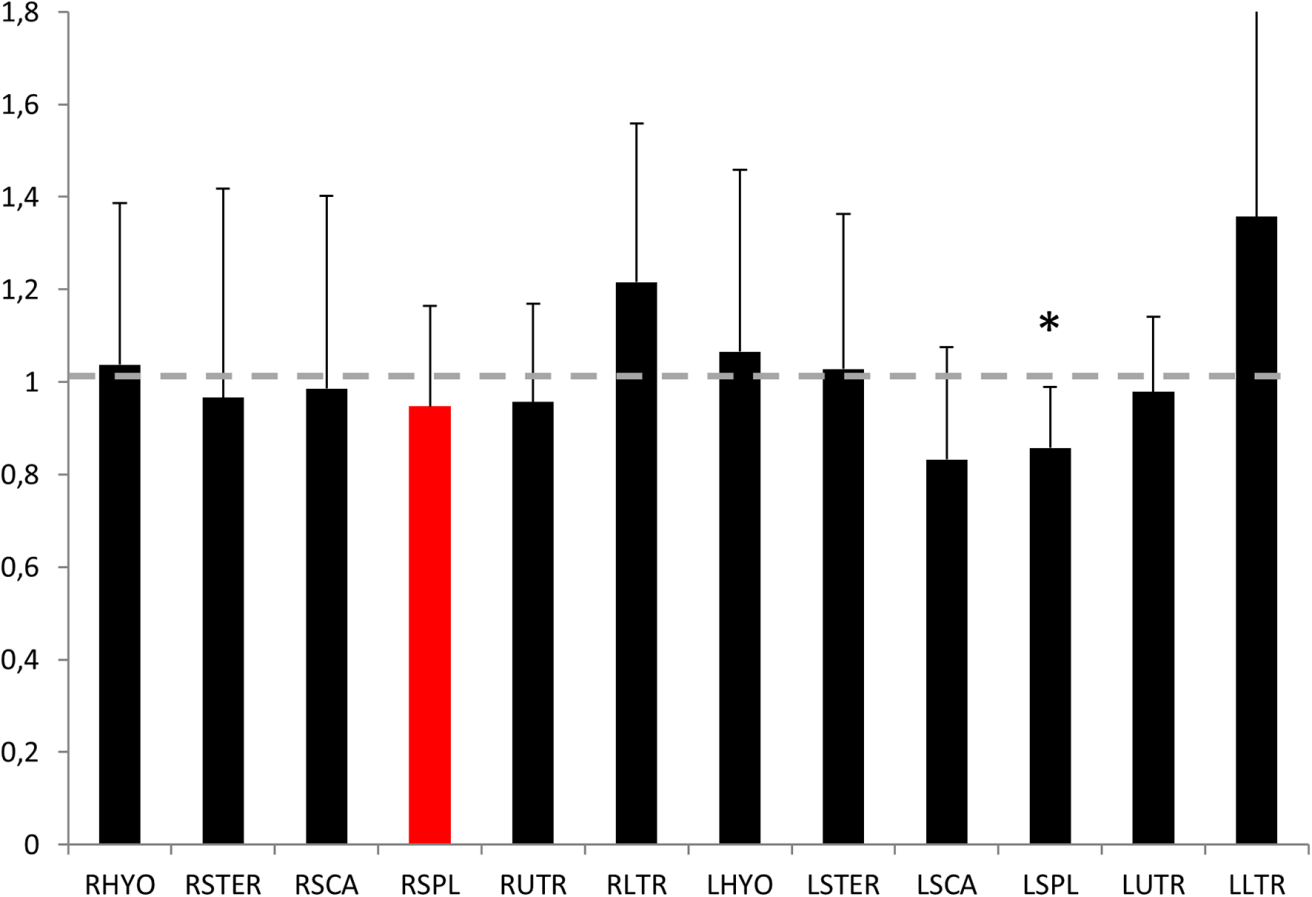
Integrated EMG amplitude for each muscle. Average and SD of the integrated EMG amplitude recorded for each muscle in the painful condition normalized relative to the baseline condition. The gray dotted line indicates the level of activity which would be comparable between conditions. The injected muscle, the right splenius capitis is highlighted in red; note the overall decreased activity of this muscle. Other muscles showed either an increase or decrease of activity across all subjects. (Right–R and Left–L: Sterno Hyoideus–HYO, Sternocleidomastoideus-STER, Anterior Scalenus-SCA, Splenius Capitis–SPL, Upper trapezius-UTR, Lower Trapezius-LTR).

Fig 3 shows the effect of the painful injection on the EMG amplitude for each muscle, integrated across the whole trial and normalized with respect to the baseline condition for all subjects. A general decrease of activity is noted for the injected muscle together with and increase or decrease of the activity of the other muscles. A significant change in the amplitude of activation was only noted for LSPL (main effect P = 0.005; BASE vs ISO = .32, BASE vs PAIN = .05, BASE vs POST = .48).

**Fig 4:**
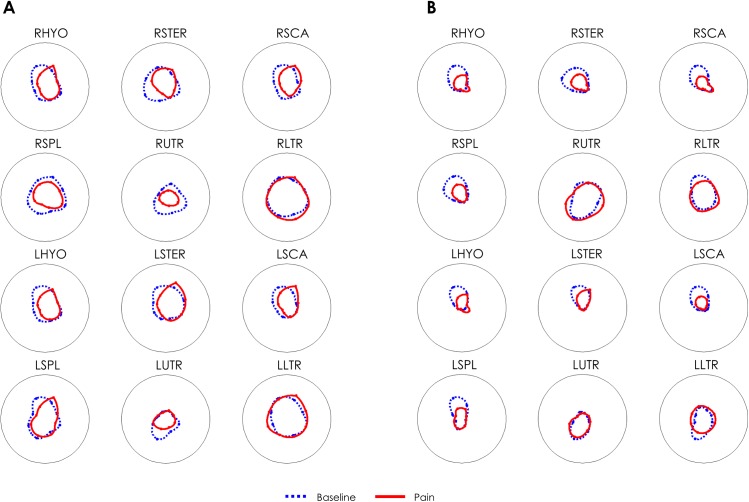
Representative EMG amplitude data from two subjects (A and B respectively). Each line represents the integrated EMG activity for each movement direction, and data is interpolated between directions for representation. Data is normalized with respect to the maximal value between BASE and PAIN for each muscle. Note the consistent decreased activity for the injected muscle (right splenius capitis) for both subjects but the variable change for the other muscles. For instance, the right upper trapezius (RUTR) shows reduced activity in the painful condition for Subject A but increased activity for subject B. (Right–R and Left–L: Sterno Hyoideus–HYO, Sternocleidomastoideus-STER, Anterior Scalenus-SCA, Splenius Capitis–SPL, Upper trapezius-UTR, Lower Trapezius-LTR).

**Fig 5:**
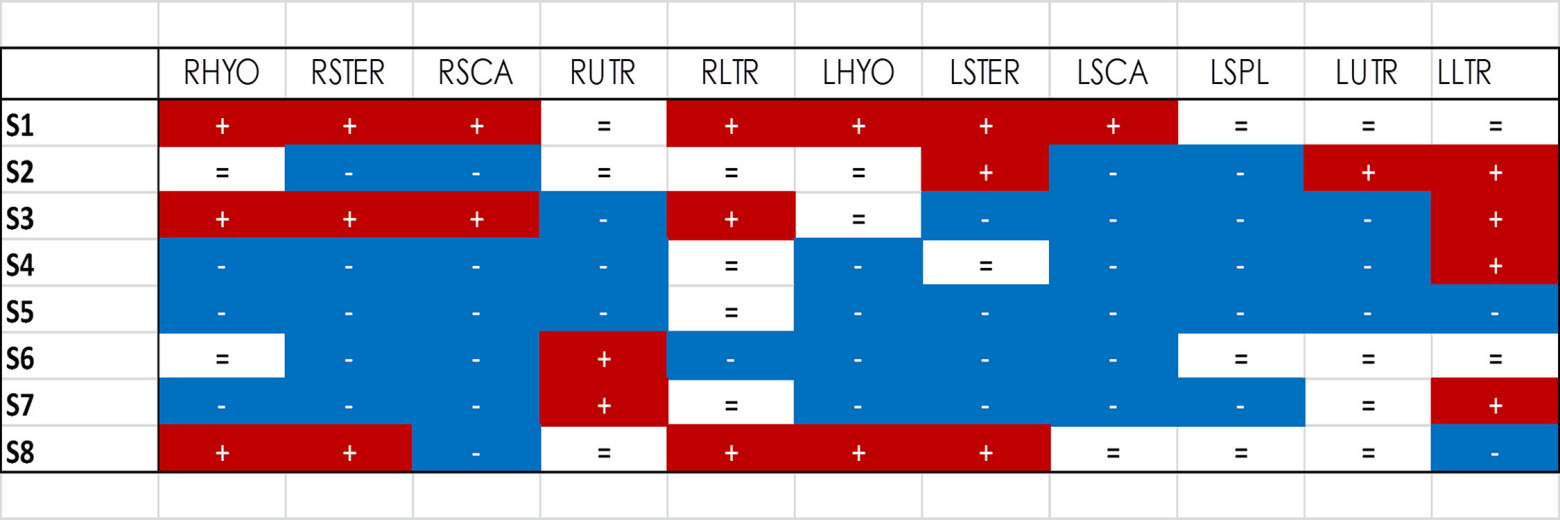
Individual variation of EMG amplitude change. Individual data for each of the eight subjects showing the direction of change in EMG amplitude of each muscle between the baseline and painful conditions. Red (+) indicates an increase of EMG amplitude in the painful condition compared to baseline, blue (-) indicates decreased EMG amplitude and white (=) indicates no change. Note the individual specific patterns of modulation of EMG amplitude. No two subjects showed the same strategy. The threshold was set to ±10% of the baseline average EMG amplitude value. (Right–R and Left–L: Sterno Hyoideus–HYO, Sternocleidomastoideus-STER, Anterior Scalenus-SCA, Splenius Capitis–SPL, Upper trapezius-UTR, Lower Trapezius-LTR).

Individual subjects, responded to the painful stimulus in a heterogeneous manner. Fig 4 shows an example of the whole muscle set of two subjects recorded during the BASE and PAIN conditions. Note that although both subjects show reduced activity of the injected muscle, the response of the synergist and antagonist muscles is subject-specific. Fig 5 illustrates the change in activation of each synergist and antagonist muscle for each subject. Note that no two subjects showed the same adaptation of muscle activity in the painful condition. In this case a threshold of 10% BASE amplitude was used to define whether the muscle activity increased, decreased or remained unchanged.

### Reconstruction quality

Four modules were sufficient to reconstruct the original EMG signal according to the chosen quality parameters (VAF: BASE 0.96±0.02, ISO 0.97±0.01, PAIN 0.96±0.03, POST 0.97±0.02). The quality of reconstruction did not show significant differences among conditions (P = 0.84); adding another module would have led to a minor increase of reconstruction quality (0.98±0.01, average across all subjects and all conditions), while the elimination of the fourth module would have led to a reduction of the VAF >3% (0.92±0.04, average across all subjects and all conditions [36,48]).

The extraction of muscle synergies was performed only on one turn although the data was recorded for longer time in order to guarantee that the turn with highest pain was always included in the recording for the painful condition. Further comparison of muscle synergies extracted from different turns of the baseline confirmed that they strategy adopted by individual subjects in the pain free condition was coherent across runs (NDP between turn 1 and turn 2 of the baseline was 0.92±0.1) and that therefore the data chosen was representative of the muscular activation in that condition.

### Motor modules similarity

The average NDP of the four motor modules extracted for all subjects for the BASE condition was 0.73±0.14. Similar results were found for the other conditions (NDP: ISO 0.74±0.16, PAIN 0.69±0.12, POST 0.76±0.13, P = 0.795). This result reveals that among different subjects, the level of similarity of motor modules was lower than within the same subject across different conditions (see below).

Compared to BASE, the ISO and POST conditions showed comparable values of average motor module similarity (n = 8; NDP: BASE vs ISO 0.91±0.09 and BASE vs POST 0.92±0.06, respectively, ISO vs POST P = 0.575) while for the PAIN condition, the level of similarity was significantly lower (n = 8; NDP: BASE vs PAIN 0.79 ±0.11, ISO vs PAIN P = 0.012, POST vs PAIN P = 0.036).

The CPA analysis revealed that for 6 out of 8 subjects, the set of motor modules of the PAIN condition compared with BASE shared a three dimensional space (i.e. one module was below threshold); for the other 2 subjects no CPAs were below threshold. For the ISO condition, 4 subjects shared three motor modules with BASE, whilst the remaining four had all the CPAs above the threshold. However the average of CPA below threshold was higher than the corresponding average for PAIN (ISO: 0.71±0.05, PAIN: 0.61±0.14). Finally, for the POST condition, modules shared a three dimensional space with BASE in only 3 subjects, while the other subjects showed a 4-dimensional shared space.

**Fig 6:**
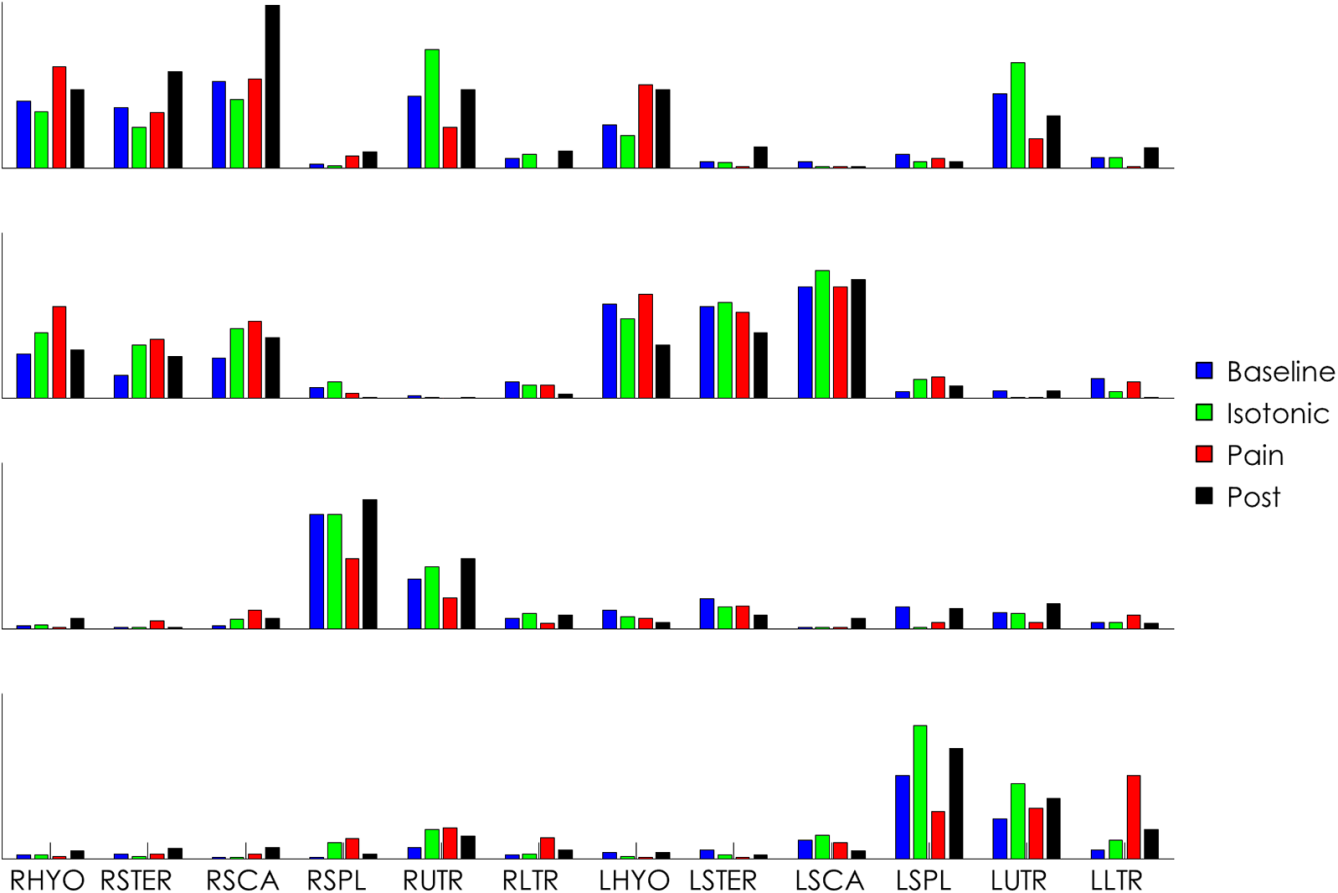
Motor modules from a representative subject. Each sub-graph represents one motor module (top-bottom: modules 1 to 4). In this example the activity of the RSPL (injected muscle) is mainly represented in module three. Note the change of activity of this muscle in the painful condition compared to the other three conditions. The painful condition is also characterized by a change of the activity of the contralateral splenius capitis (also reduced) and an increase of activity of the contralateral lower trapezius (module 4). The accomplishment of the task is also achieved by fine tuning of the activation of most of the other muscles.

As an example, motor modules from one subject are represented in Fig 6. The modules in all conditions are ordered to match the BASE condition.

The correlation of the activation signals, on the other hand, showed homogenous values across conditions (zero-lag normalized cross correlation values: BASE vs ISO: 0.73±0.16, BASE vs PAIN: 0.70±0.09, BASE vs POST: 0.69±0.10, P = 0.61).

### Cross reconstruction

**Fig 7:**
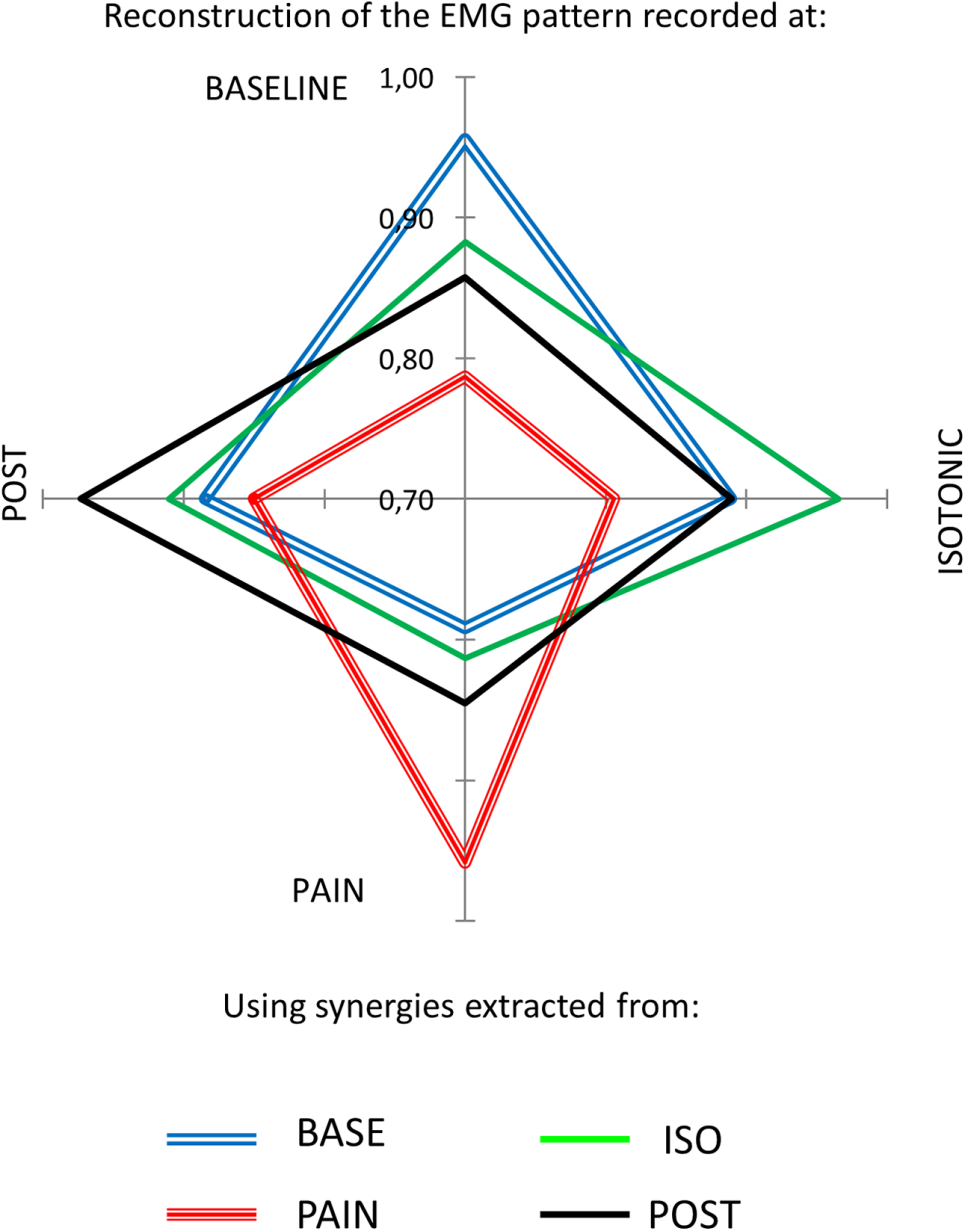
Cross-reconstruction performance. Cross-reconstruction performance for baseline (blue, double line), isotonic (green), pain (red, triple line) and post (black) conditions. To test if the painful sensation would influence the muscle synergies, the muscle weightings from one condition were used to reconstruct (by means of non-negative reconstruction [34]) the EMG from all the conditions while the activation signals were free to vary. This directly excludes the possibility that the baseline weighting coefficients are maintained and recruited with a different temporal behavior. The reconstruction quality was reported as Variance Accounted For. The results are reported in the figure: on each axis branch the reconstruction quality of all the conditions with respect to one condition is reported—e.g. the upper axis branch represents the VAF index measured for the reconstruction of the four conditions with the modules extracted from baseline. The highest performance is obtained with the modules extracted from the same condition.

The cross reconstruction of each condition with modules from other conditions confirmed the presence of a shared structure of motor control across the BASE, ISO and POST conditions, but not for the PAIN condition (see Table 3). In this case the muscle weightings from one condition were retained and used to reconstruct all four conditions and the average results are shown in Fig 7 where the behaviour of PAIN synergies (red line) reflects the results reported for the similarity indices (i.e. that the PAIN synergies are different with respect to those extracted from the other conditions).

**Table 3:** Cross-reconstruction Variance Accounted For (VAF) index. The reconstruction quality was highest on the diagonal of the matrix (i.e. when the EMG of one condition was reconstructed with its own modules, in bold). The cross-reconstruction quality was lower when using motor modules from any condition to reconstruct the EMG from the PAIN condition and vice versa (i.e. when the modules from the PAIN condition were used to reconstruct the EMG of other conditions).

EMG\modules	BASE	ISO	PAIN	POST
BASE	**0,96±0,02**	0,89±0,11	0,79±0,17	0,89±0,12
ISO	0,88±0,19	**0,97±0,01**	0,81±0,15	0,91±0,17
PAIN	0,79±0,19	0,81±0,17	**0,96±0,03**	0,85±0,16
POST	0,86±0,08	0,89±0,08	0,84±0,16	**0,97±0,02**

It is possible that the reduced reconstruction quality of PAIN EMG with BASE modules could be solely attributed to altered activity of the injected muscle (and not to the reorganization of the other muscles). To test this hypothesis, the cross-reconstruction was repeated on a reduced dataset, in which the RSPL EMG signal was removed. The components relative to RSPL were removed from the motor modules of the BASE condition. The value of VAF was, however, in line with the previous result (0.80±0.14) confirming that the reorganization of muscular activity occurred not only because of altered activity of the injected muscle.

In summary, this study examined the effects of experimentally induced muscle pain on multi-muscle control of neck movements during an aiming task. The results show that groups of muscles are recruited together on a subject-specific basis and that the modular nature of motor control is maintained in the presence of acute muscle pain (e.g.in all of the subjects the dimensionality of control was unchanged with pain and for two subjects the motor modules extracted in PAIN shared a 4-dimensional space with those extracted in the BASE condition). Despite a change in the activation of various muscles in the painful condition, the kinematic output was unchanged.

Although pain can impair motor output [13,50], several studies have shown that in presence of goal-oriented submaximal tasks, the kinematic output may be unchanged [34,51–53]. Muscular adaptation (i.e. redistribution of the activity within the same muscle or across synergistic muscles) occurs to maintain motor output unaltered but with modified muscle load [54].

The performance of head movement requires fine and accurate motor control, which is achieved by exploiting the elevated number of muscular actuators available in the neck region in a flexible and precise manner. Previous studies have shown that the control of neck movement may not follow intuitive, dynamically-sound rules of muscle recruitment [55], unless the task requires a moderate to high level of force and the mechanical constraints are strict [19]. A possible implication of this is that the CNS may be able to span a vast landscape of motor control solutions for a given task by means of preferential muscle couplings (as was shown in this experiment) and that the muscle associations may vary across individuals as long as the dynamic output is distant from the maximal achievable in a given condition and a suboptimal but satisfactory motor solution is available [21]. The presence of individualized motor modules has been previously associated with the continuous refinement of the internal representation of the biomechanics of the body by the CNS [33]. It is also worth noting that a more stereotyped motor control can be found in other tasks where the energy consumption or net force output are crucial (e.g. locomotion) [23,46].

The presence of similar motor output resulting from the combination of different patterns of muscular activation is compatible with the idea that the control strategy is not dictated by biomechanical constrains but rather by muscle groupings that have neural origin, however it has to be kept in mind that the task not being completely unconstrained (the subject were requested to reach the target in one single movement and avoid further corrective actions) and the target area not spanning the whole space of possible movements of the head may have promoted evidences for motor control regularities.

The main effect of experimental muscle pain is a temporary decrease of EMG amplitude of the painful muscle [56,57], together with compensatory strategies from synergistic muscles [12,57,58]. In the current experiment, a general decrease of the EMG amplitude in the injected muscle was observed together with redistribution (decrease/increase) of the activation of other muscles, in a subject-specific manner, whilst the control strategy was maintained modular. The recruitment of modules specific to the painful condition is reflected by the reduced cross reconstruction quality and is in line with previously reported results on the upper limb [17]. The cross reconstruction is used to directly test whether muscle weightings extracted from one condition can be combined to generate the activation pattern seen in another condition (coupled with a different set of activation signals); from the results of our cross reconstruction tests it emerged that the muscle weightings of non-painful conditions are not suitable to reconstruct the activation patterns of the painful condition. On the other hand, the results on cross correlation of the activation signals themselves confirmed that the difference is mainly in muscle weightings.

Hodges and Tucker [59] proposed a theory which predicts heterogeneous adaptation in motor control in response to pain. Recent studies [17,60] have linked the individual response to a painful stimulus to an increase of motor control variability, related to the idea that the CNS would explore different biomechanical solutions to the motor task in order to trade off the preservation of the “damaged” tissue with a sufficiently effective accomplishment. Our results support this theory showing that the responses to similar painful stimuli are subject-specific and not stereotyped and are also in line with those reported on the influence of acute pain in muscle synergies during locomotion, where the authors found subject specific adaptation of some synergies to the painful stimulus [40].

The activation signals are associated with the timing of the execution of the movement; this feature has shown robust consistency across different timely precise tasks [23,38,46,61,62]. However it was also shown that the CNS is able to dynamically tune the activation signals in case of sudden mechanical perturbations [63]. In the current study, the activation signals showed a comparable level of cross correlation across conditions, whilst the motor modules showed a lower level of similarity in the PAIN condition. Moreover, one of the modules changed for most of the subjects in the PAIN condition.

It is not possible to exclude that, given the relatively small anatomical region examined, the dimension of the electrodes and the spatial distribution of the muscles investigated, part of the common features revealed by the study of motor modules could be imputed to EMG cross-talk. It is also true that while performing a goal-oriented task without any previous specific training it is very complex (if not practically impossible) to selectively activate individual muscles while maintaining a comparable mechanical output. On the other hand, the electrical activity of different muscles changed during the PAIN condition, whether or not the injected muscle (RSPL) was removed from the dataset. This can be interpreted as an individual redistribution of muscular load and thus as a suggestion that the eventual presence of cross-talk could be considered negligible with respect to the signal generated by the individual muscles. It has been shown [6,64], that pain induces a reorganization of activity across superficial and deep neck muscles. Deep neck muscles were not investigated in this study but it can be hypothesized that a further reorganization may occur at that level.

Subjects were asked to perform the aiming either in clockwise or counterclockwise sequence. Although other studies on reaching/aiming tasks reported randomized rather than continuous sequences [17,34], in our pilot experiments the subjects were not able to see the target numbers without causing movement of the head. Thus the sequenced aiming solved the problem of trajectory and endpoint uncertainties and corrections.

## Conclusions

Experimentally induced muscle pain induced a reorganization of motor control of the neck region at a multi-muscular level, without an appreciable change of the task kinematics. Although the changes in the motor strategy may show some distinct characteristics across individuals, the majority of subjects showed decrements in similarity of motor modules for the painful condition compared with control conditions. This is in agreement with the hypothesis that the individual muscle weights are adjusted in a dynamic task-dependent manner under the influence of afferent input. The cumulative changes of activity among different muscles suggest a dynamic modulation in the activity of individual muscles to cope with the painful stimulus in order to correctly accomplish the requested task.
